# Management of Extrahepatic Portal Vein Obstruction With Spleno-Adrenal Shunt

**DOI:** 10.7759/cureus.81699

**Published:** 2025-04-04

**Authors:** Brijesh Gyadari, Manjesh K A, Ashwini Dutt, Kishore Abuji, Venu Bhargava M

**Affiliations:** 1 Department of General Surgery, Employees' State Insurance Corporation (ESIC) Medical College, Hyderabad, IND; 2 Department of Vascular Surgery, Sree Chitra Tirunal Institute for Medical Sciences and Technology, Thiruvananthapuram, IND; 3 Department of Surgical Gastroenterology, Employees' State Insurance Corporation (ESIC) Medical College, Hyderabad, IND

**Keywords:** extrahepatic portal hypertension, idiopathic portal hypertension, portal thrombosis, spleno-adrenal shunt, splenorenal shunt

## Abstract

Extrahepatic portal hypertension is the hypertension of the portal venous system in the absence of liver cirrhosis, and variceal bleeding is its commonly seen complication. Long-standing portal hypertension will have the risk of symptomatic hypersplenism, portal biliopathy, growth failure, and ectopic varices. Portosystemic shunt surgery can more effectively manage these complications than medical and endoscopic management. Conventional portosystemic shunts, especially the proximal splenorenal shunt (PSRS), are preferred over unconventional shunts, as the latter will have increased procedural complexities and increased postoperative morbidity. Though spleno-adrenal shunt (SAS) surgery is an unconventional type that offers an excellent alternative to PSRS with equal outcomes. Here, we present a case of a 21-year-old male who presented with a mass in the abdomen since childhood, with one episode of hematemesis. Upon thorough clinical examination, laboratory and radiological investigations made a diagnosis of extrahepatic portal hypertension with symptomatic hypersplenism, following which the patient underwent splenectomy, followed by SAS.

## Introduction

Extrahepatic portal vein obstruction (EHPVO) is the most important cause of non-cirrhotic portal hypertension and upper gastrointestinal bleeding, particularly in third-world countries [1-3]. Portal vein cavernous transformation (PVCT) is one of the hallmarks of EHPVO, where the portal vein is transformed into a cavernoma by developing extensive collaterals involving pericholecystic, paracholedochal, and pancreaticoduodenal regions, which leads to portal hypertension, esophagogastric varices, formation of ectopic varices, and portal biliopathy [2,4]. Portosystemic shunt surgery, which decompresses the portal system, is frequently performed in the treatment of variceal bleeding and portal hypertension, especially with non-cirrhotic type, as it has minimal recurrence when compared to medical and endoscopic treatments. Although conventional portosystemic shunt surgery, particularly proximal splenorenal shunt (PSRS), is commonly performed in non-cirrhotic portal hypertension, spleno-adrenal shunt (SAS) is preferred when there is a dilated adrenal vein in cases of difficulties in isolation of the renal vein. Here, we present a case of EHPVO successfully managed using SAS [4,5].

## Case presentation

**Figure 1:**
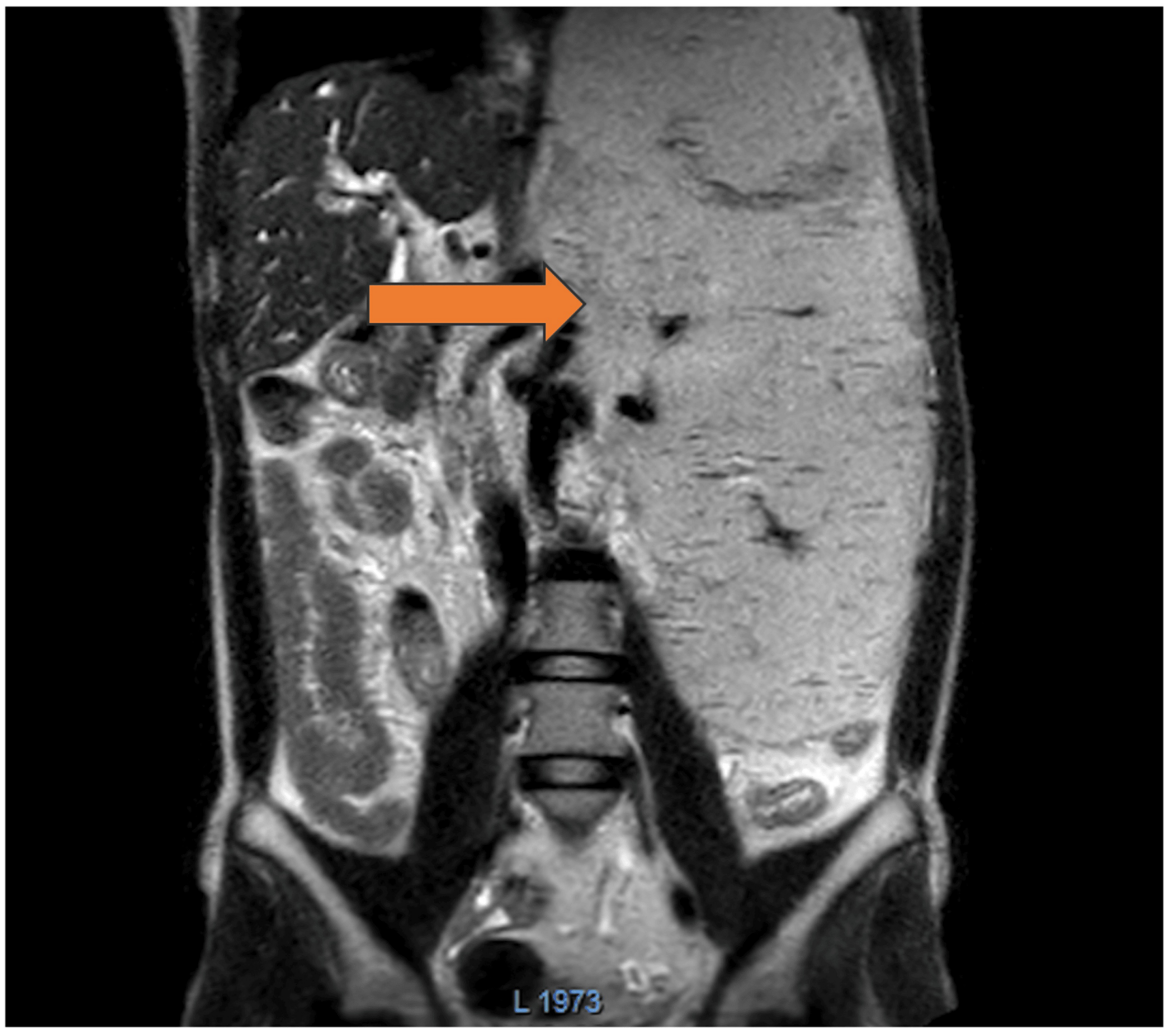
Coronal view of MRI of the abdomen showing massive splenomegaly (arrow mark).

A 21-year-old male presented with left upper abdominal pain and a lump since childhood, which was noticed incidentally by his father. The patient reported a history of hematemesis six years ago, managed through endoscopic variceal ligation (EVL). An abdominal examination revealed a mass likely of splenic origin, and blood investigations suggested features of hypersplenism, with normal liver function tests and blood coagulability. An ultrasound of the abdomen indicated that the spleen was enlarged to 21 cm, with normal liver size. Doppler ultrasound showed attenuation of the portal vein with multiple collaterals in the periportal region, suggestive of chronic thrombosis. CT portal-venography revealed multiple collaterals in the peri-splenic area, with a dilated splenic vein measuring 1 cm in calibre and a renal vein also measuring 1 cm, accompanied by portal cavernoma. The adrenal vein was 8 mm, with mild retroperitoneal fat stranding observed. MRI of the abdomen was done outside, which showed an enlarged spleen (Figure 1). Upper gastrointestinal endoscopy revealed large three-column varices, which had been managed by EVL six years prior.

**Figure 2:**
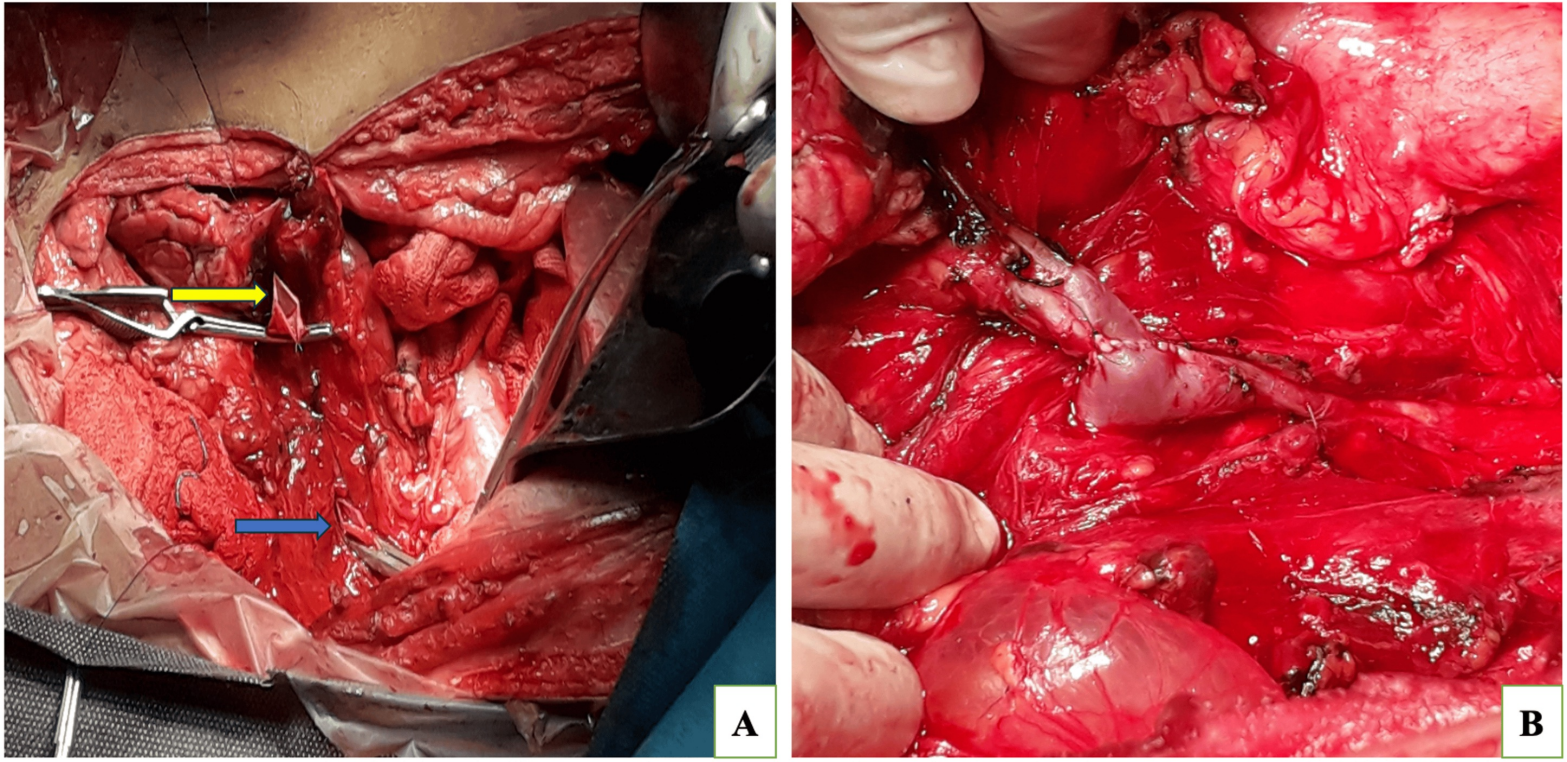
(A) Intraoperative image showing the cut end of the splenic vein (yellow arrow) and dilated left adrenal vein (blue arrow). (B) Intraoperative image showing anastomosis between the splenic (end) and adrenal vein (side).

The patient was prepared for splenectomy, followed by a splenorenal shunt, for which he was vaccinated two weeks before the surgery. The abdomen was opened in layers through the left Makuuchi incision, and the peri-splenic collaterals were identified and divided. The splenic artery was identified and divided as well. The splenic vein was recognized and freed from the pancreas by ligating and dividing its tributaries (Figure 2A). Splenectomy was performed, after which a shunt was created between the splenic vein and the left adrenal vein through an end-to-end anastomosis, given the dilated adrenal vein. The preoperative plan was to make a splenorenal shunt. During surgery, we identified a dilated adrenal vein with multiple adhesions around the renal vein, so we decided on an intraoperative SAS. Doppler showed free flow from the splenic vein to the renal vein via an adrenal vein (Figure 2B). The postoperative course was uneventful, and the patient was discharged on postoperative day five. At 36 months post shunt, the patient is doing well, with no upper gastrointestinal bleed or jaundice. The patient is under regular follow-up.

## Discussion

Extrahepatic portal hypertension is defined as extrahepatic hypertension of the portal venous system in the absence of liver cirrhosis. Patients with long-term complications such as recurrent variceal bleeding and symptomatic hypersplenism are considered high-risk groups, and they often require portosystemic shunt surgery more than medical and endoscopic management [6]. Portosystemic shunt surgery offers the most effective and durable treatment for the management of extrahepatic portal vein obstruction, especially in high-risk groups, with few complications like bleeding, thrombosis, and hepatic encephalopathy. They lower the elevated portal pressure by decompressing the portal system, unlike other lesser invasive methods to control variceal bleeding, such as endoscopic sclerotherapy and endoscopic band ligation, and also preserve adequate flow through the liver to prevent encephalopathy and ascites [7].

In 1947, Robert Linton published a paper regarding five patients who underwent splenectomy and proximal splenorenal venous anastomosis [8]. In 1989, Mazariegos and Reyes in Pittsburgh described a modification of the splenorenal shunt by using the adrenal vein as the inflow vessel into the renal vein with long-term shunt patency and minimal morbidity [9]. In 2012, Gu et al. in Shanghai proved that spleno-adrenal shunt was non-inferior to PSRS in terms of reduction in post-shunt portal hypertension, long-term patency, variceal regression, and reversal of hypersplenism [4]. Linton's shunt (splenectomy with PSRS) does not need any type of graft and has a very minimal risk for postoperative encephalopathy or mortality [3]. Anatomical or pathological abnormalities in the splenic vein make splenorenal anastomosis challenging and sometimes compulsions to go for other unconventional shunts, such as Rex bypass or selective portocaval shunts. These unconventional shunts require either natural or synthetic grafts and are usually associated with increased procedural complexities as well as increased rates of complications, thus making their use limited. However, the spleno-adrenal shunt is an exception, as it offers comparable efficacy to PSRS while avoiding the drawbacks of other unconventional shunts [3].

The adrenal vein is considered a suitable conduit for splenorenal anastomosis due to some factors. The use of a natural conduit is superior to that of a prosthetic graft in terms of reduced risk of infection and thrombosis. The left adrenal vein lies in the area of dissection during isolation of the left renal vein in surgery, and its drainage into the left renal vein with an optimal anatomic angle makes a tension-free anastomosis. Furthermore, using the adrenal vein deflects the need for direct vascular anastomosis into the renal vein, eliminating the requirement for renal vein clamping, which could potentially cause postoperative thrombosis [10]. The distal and proximal splenorenal/adrenal shunts have shown equal efficacy in preventing bleeding from gastroesophageal varices, while the proximal splenorenal/adrenal shunt is more effective in reversing hypersplenism clinically and prevents ectopic variceal bleeding as well as portal biliopathy [10].

## Conclusions

Portal cavernoma transformation in EHPVO, which is the most important cause of noncirrhotic portal hypertension, leads to portal hypertension, variceal bleeding, and other long-term complications. Portosystemic shunt surgeries are effective and durable treatments in the management of noncirrhotic portal hypertension and its long-term complications. The Linton's shunt, a type of conventional shunt, is the most accepted surgery worldwide, whereas unconventional portosystemic shunts are not preferred, as they are associated with increased procedural complexities and postoperative morbidity, but SAS makes an exception to this drawback. SAS is an effective alternative to PSRS with equal outcome for managing EHPVO, especially in cases with a dilated adrenal vein. It offers a tension-free anastomosis, avoids renal vein clamping, and reduces complications while effectively controlling portal hypertension and hypersplenism.

## References

[1] Sarin SK, Agarwal SR (2002). Extrahepatic portal vein obstruction. Semin Liver Dis.

[2] Warren WD, Millikan WJ Jr, Smith RB 3rd (1980). Noncirrhotic portal vein thrombosis. Physiology before and after shunts. Ann Surg.

[3] Prasad AS, Gupta S, Kohli V, Pande GK, Sahni P, Nundy S (1994). Proximal splenorenal shunts for extrahepatic portal venous obstruction in children. Ann Surg.

[4] Gu S, Chang S, Chu J, Xu M, Yan Z, Liu DC, Chen Q (2012). Spleno-adrenal shunt: a novel alternative for portosystemic decompression in children with portal vein cavernous transformation. J Pediatr Surg.

[5] Limbu Y, Regmee S, Maharjan DK, Thapa PB (2021). Spleno-adrenal shunt: a feasible alternative to splenorenal shunt in extrahepatic portal hypertension. J Kathmandu Med Coll.

[6] Aydin U, Yazici P, Kilic M (2007). Porto-systemic shunt using adrenal vein as a conduit; an alternative procedure for spleno - renal shunt. BMC Surg.

[7] Malviya NK, Behari A, Kumar A, Kapoor VK, Saxena R (2022). Unconventional shunts in extrahepatic portal venous obstruction—a retrospective review. J Clin Exp Hepatol.

[8] Linton RR, Jones CM, Volwiler W (1947). Portal hypertension: the treatment by splenectomy and splenorenal anastomosis with preservation of the kidney. Surg Clin North Am.

[9] Mazariegos GV, Reyes J (1998). A technique for distal splenoadrenal shunting in pediatric portal hypertension. J Am Coll Surg.

[10] Gupta S, Venkata Srinivas G, Chandrasekar AS, Kalayarasan R, Pottakkat B (2019). Splenoadrenal shunt for noncirrhotic portal hypertension. Indian J Surg.

